# Amine–Borane Dehydropolymerization: Challenges and Opportunities

**DOI:** 10.1002/chem.201804592

**Published:** 2018-12-27

**Authors:** Annie L. Colebatch, Andrew S. Weller

**Affiliations:** ^1^ Department of Chemistry University of Cambridge Lensfield Road Cambridge CB2 1EW UK; ^2^ Department of Chemistry University of Oxford Mansfield Road Oxford OX1 3TA UK

**Keywords:** amine–borane, catalysis, dehydrogenation, dehydropolymerization, polyaminoborane

## Abstract

The dehydropolymerization of amine–boranes, exemplified as H_2_RB⋅NR′H_2_, to produce polyaminoboranes (HRBNR′H)_*n*_ that are inorganic analogues of polyolefins with alternating main‐chain B−N units, is an area with significant potential, stemming from both fundamental (mechanism, catalyst development, main‐group hetero‐cross‐coupling) and technological (new polymeric materials) opportunities. This Concept article outlines recent advances in the field, covering catalyst development and performance, current mechanistic models, and alternative non‐catalytic routes for polymer production. The substrate scope, polymer properties and applications of these exciting materials are also outlined. Challenges and opportunities in the field are suggested, as a way of providing focus for future investigations.

## 1 Introduction

**Scheme 1:**
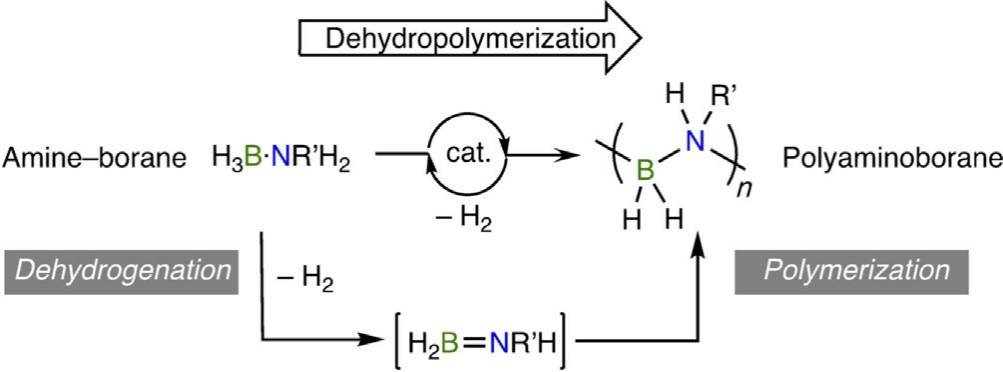
Amine–borane dehydropolymerization.

The synthesis of polyaminoboranes (HRBNR′H)_*n*_, polymers with alternating B−N main‐chain units, has attracted considerable interest since the groundbreaking report in 2008 by Manners and co‐workers of the catalyzed dehydropolymerization of H_3_B⋅NMeH_2_ to produce high molecular weight, soluble, polymers (Scheme 1).^1^, ^2^ In the ten years since, considerable progress has been made in understanding and developing this methodology for the synthesis of main‐group polymeric materials.^3^, ^4^, ^5^, ^6^, ^7^, ^8^ Challenges remain, however, in both fundamental mechanistic understanding and translating this to the production of tailored new materials.

In addition to their role as pre‐ceramics for B−N containing materials,^9^, ^10^, ^11^ much of the interest surrounding polyaminoboranes stems from the BN unit being an isostere of C−C, and thus the formal relationship to simple polyolefins. These industrially and societally important materials are manufactured on a huge scale,^12^ often by the coordination/insertion polymerization of simple alkene monomers, such as propene. The development of tailored materials properties by mechanism‐led design has had (organometallic) catalyst design, synthesis and function at the forefront.^13^ In contrast to polyolefins, the monomer units for polyaminoboranes, primary aminoboranes (HRB=NR′H), are thermally unstable with respect to uncontrolled oligomerization by head‐to‐tail intermolecular B−N bond formation, and have only been observed as transient species^14^, ^15^ or trapped by coordination to a transition metal fragment.^16^, ^17^ Hence, the production of well‐defined polymeric B−N materials by a dehydropolymerization strategy is complicated by the fact that two elementary processes are required—dehydrogenation of amine–boranes (H_2_RB⋅NR′H_2_) to give reactive aminoboranes and subsequent controlled^18^ polymerization through head‐to‐tail B−N bond formation (Scheme 1).

Amine–boranes are formally isoelectronic to alkanes, for which closely related non‐oxidative alkane dehydrogenation is endothermic, requiring an H_2_ acceptor and/or high temperatures.^19^ In contrast, amine–borane dehydrogenation is enabled kinetically by the polarity of the B−H and N−H bonds,^20^ and thermodynamically by typically being exothermic.^21^ Thus, although catalysis or an acceptor are not required for dehydrogenation or subsequent polymerization of amine–boranes, reaction temperature and product distributions^22^ can be strongly influenced by catalyst identity.

There are now many catalysts known that are capable of the very efficient dehydrogenation of amine–boranes, including transition‐metal complexes, s‐block compounds and main‐group Lewis acids.^5^, ^6^, ^7^ The challenge in catalytic amine–borane dehydropolymerization, then, is not to effect dehydrogenation but rather to do so with a high degree of control to afford well‐defined polymeric products in the subsequent B−N coupling steps, in an overall atom efficient manner and with high compositional purity. This Concept article serves to outline the current position of amine–borane dehydropolymerization, and identify key challenges and opportunities that the community may find useful to consider as the field develops. For brevity, closely related phosphine–borane dehydrocoupling is not discussed in detail, but concepts and catalysts developed in either field serve as useful comparisons.^5^, ^23^, ^24^

## 2 Catalysts

### 2.1 Catalyst overview

**Figure 1:**
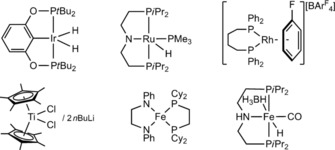
Examples of catalysts for dehydropolymerization.

Pioneering studies of amine–borane dehydropolymerization utilized the IrH_2_(POCOP) (POCOP=κ^3^‐1,3‐(OP*t*Bu_2_)_2_C_6_H_3_) catalyst initially used by Heinekey and Goldberg,^25^, ^26^ and studied in detail by Manners (Figure 1).^1^, ^27^ Since then, the number of catalysts capable of effecting such polymerizations has grown considerably, with homogeneous and heterogeneous examples now spanning the periodic table.^5^, ^6^, ^7^ We will focus on homogeneous systems here, and selected examples are shown in Figure 1. Examples of early and mid‐transition‐metal catalysts include Manners’ TiCl_2_Cp*_2_/2×*n*BuLi system,^28^ and Kawano's Mn(η^5^‐C_5_H_5_)(CO)_3_ and Cr(η^6^‐C_6_H_6_)(CO)_3_ complexes under photolytic conditions.^29^ Later transition‐metal catalysts are most common, especially those of Rh and Ru, and earth abundant systems have been developed, primarily based on iron, such as Baker's Fe(PhNCH_2_CH_2_NPh)(Cy_2_PCH_2_CH_2_PCy_2_),^30^ Manners’ photochemically activated [FeCp(CO)_2_]_2_,^31^ Schneider's FeH(CO){N(CH_2_CH_2_P*i*Pr_2_)_2_}^32^ and the related FeH(BH_4_)(CO){NH(CH_2_CH_2_P*i*Pr_2_)_2_} system employed by Beweries.^33^ Bidentate and pincer ligands feature heavily in the known catalyst systems, in particular “POCOP”,^1^, ^25^ “PNP”,^32^, ^33^, ^34^, ^35^ and “POP”^36^, ^37^, ^38^ constructs.

Transition‐metal‐free catalysis is less well developed, and we mention them here briefly before turning attention to metal‐catalyzed processes. Although several main‐group^39^, ^40^, ^41^ and frustrated Lewis pair (FLP) systems^42^, ^43^, ^44^ have been reported to catalyze the dehydrocoupling of amine–boranes, no examples of well‐defined polymer formation yet exist, though production of chain‐branched (H_2_BNH_2_)_*n*_ using a stoichiometric amount of the FLP system P*t*Bu_3_/B(C_6_F_5_)_3_ 45 has been reported, whereas dimethylxanthene‐based FLPs slowly oligomerize (dimerize) H_3_B⋅NMeH_2_.^43^ Base‐promoted anionic chain‐growth dehydropolymerization of H_3_B⋅NH_3_ forms well‐defined short‐chain oligomers,^46^ and ionic liquids have been used to promote transition‐metal‐based dehydropolymerization, in which the associated anion was shown to have a profound effect on selectivity.^47^

**Scheme 2:**
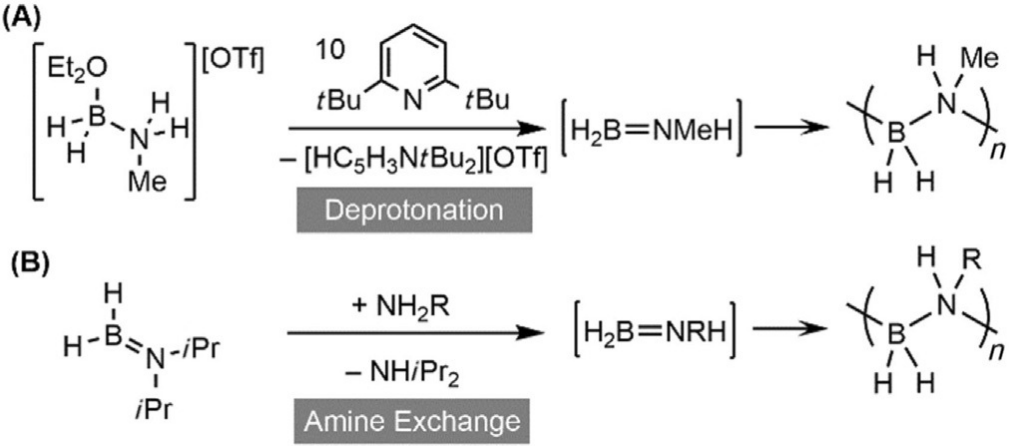
Catalyst‐free methods of polyaminoborane formation. **(A)** Deprotonation of boronium cations. **(B)** Amine exchange of *i*Pr_2_N=BH_2_.

Stoichiometric deprotonation of amine–boronium cations [MeH_2_N⋅BH_2_(OEt_2_)]^+^ produces low molecular weight (H_2_BNMeH)_*n*_, <5000 g mol^−1^, via the generation of transient H_2_B=NMeH, Scheme 2 A.^14^ Recently, a metal‐free route to generate high molecular weight polyaminoboranes has been reported using *i*Pr_2_N=BH_2_ as a {BH_2_} transfer agent in reactions with primary amines (NR′H_2_) at −40 °C to form H_2_B=NR′H in situ, Scheme 2 B; for example: (H_2_BN(C_3_H_5_)H)_*n*,_ *M_n_*=483 000 g mol^−1^, *Đ*=1.2.^48^ Both these metal‐free processes likely operate through head‐to‐tail B−N bond formation,^49^ and the large difference in characteristics of the (H_2_BNMeH)_*n*_ formed in these two systems (*M*_w_ <5000 vs. 201 000 g mol^−1^), despite deriving from a common aminoborane intermediate, H_2_B=NMeH, shows that conditions of concentration, temperature and solvent are likely crucial. This suggests further opportunities for optimization of transition metal catalyzed processes using such straightforward parameters.^27^

### 2.2 Catalyst development

The iterative development of new generation catalysts based on specific performance parameters of the polymer they produce, such as molecular weight (*M_n_*), dispersity (*Đ*), degree of chain branching and compositional purity has not yet been widely applied in amine–borane dehydropolymerization. Surprisingly, despite the key role of the IrH_2_(POCOP) catalyst in the area no derivatives of this system have been investigated, although there is a note that 3,5‐COOMe substituted derivatives show enhanced activity for the dehydrogenation of hydrazine borane.^50^ This lack of data is in contrast to widely explored alkane dehydrogenation where ligand derivatization has been shown to strongly modify catalyst performance.^19^ However, catalyst variations have been studied in a number of other systems, providing tentative steps towards catalyst structure activity relationships.

This has been most thoroughly investigated in Weller's cationic systems, based upon {Rh(chelating diphosphine)}^+^. For example [Rh{R_2_P(CH_2_)_*n*_PR_2_}]^+^ (*n*=1–5, R=Ph, *i*Pr) provide information on the first‐formed species in dehydropolymerization,^16^, ^51^, ^52^ whereas P,O,P‐Xantphos‐R ligands have been used to delineate effects of phosphine substituent (R=Ph, Et, *i*Pr, *t*Bu) and catalyst charge, which lead to significant changes in mechanism and the nature of the polymer produced.^36^, ^37^ The {Rh(Xantphos‐Ph)}^+^ system is noteworthy in that it offers control over polymer molecular weight by manipulation of catalyst loading and reaction conditions such as solvent or hydrogen pressure.^37^

Schneider has compared the pre‐catalysts RuH(PMe_3_){N((CH_2_)_2_P*i*Pr_2_)_2_} and RuH_2_(PMe_3_){NR((CH_2_)_2_P*i*Pr_2_)_2_} (R=H, Me) in the dehydropolymerization of H_3_B⋅NH_3_, wherein subtle changes in catalyst structure resulted in mechanistic changes based on experimental and computational findings.^35^ Dehydrogenation of ammonia‐borane by the cobalt catalysts CoH{E(CH_2_CH_2_PPh_2_)_3_} produces (H_2_BNH_2_)_*n*_ when E=P, but when E=N two equivalents of H_2_ are liberated and B‐(cyclotriborazanyl)amine–borane (BCTB), borazine, and polyborazylenes are obtained, which was attributed to the coordinative flexibility of the NP_3_ ligand.^53^ Further understanding of how catalyst structure influences mechanism will be essential for understanding and optimization of reactivity for the tailored production of polymeric materials.

### 2.3 Catalyst performance

Measures to compare catalyst efficiencies have either focused on the rate of hydrogen gas production, which is a proxy for the rate of polymer formation as it measures dihydrogen release from amine–boranes to form aminoboranes (Scheme 1); or the molecular weight (*M_n_*) and dispersity (*Đ*) of the polymeric material obtained, as generally measured by gel permeation chromatography (GPC) using polystyrene calibration standards. Such relative measurements may overestimate the actual degree of polymerization, as discussed in Section 5.

Table 1 highlights examples of the molecular weights so far obtained by catalyzed and non‐catalyzed methods. This ranges from polymeric/oligomeric material that is less than 5000 g mol^−1^, up to much higher degrees of polymerization, for example, 191 000 g mol^−1^. Despite this relatively wide distribution, the basic understanding of how the degree of polymerization/*Đ*/chain‐branching influences the bulk materials performance of polyaminoboranes is lacking, such as: rheological, solubility, mechanical (e.g., yield and tensile stress, casting properties) and pre‐ceramic properties. Thus, the demarcations that define desirable differences in such properties have not been developed. In lieu of such empirical data, we suggest the following loose *M_n_* definitions (for GPC data calibrated relative to polystyrene standards) to describe the degree of polymerization in polyaminoboranes:

**Table 1:** Selected, representative molecular weight and dispersity data for (H_2_BNMeH)_*n*_ prepared using different catalysts and methods.

Catalyst	[H_3_BNMeH_2_]:[cat]	*M_n_* [g mol^−1^]^[a]^	*Ð*	Ref.
[Rh{Xantphos‐*i*Pr}H_2_(H_3_BNMe_3_)][BAr^F^_4_]	500	9500	2.8	[36]
[Rh{Xantphos‐Ph}{H_2_BNMe_3_(CH_2_)_2_ *t*Bu}][BAr^F^_4_]^[b]^	500	23 000 (2800)	2.1 (1.8)	[37]
[Rh{Ph_2_P(CH_2_)_3_PPh_2_}(η^6^‐C_6_H_5_F)][BAr^F^_4_]^[b,c]^	200	28 000 (4700)	1.8 (2.0)	[16, 54]
FeH(BH_4_)(CO){NH(CH_2_CH_2_P*i*Pr_2_)_2_}	20	35 000	2.2	[33]
Rh{Xantphos‐*i*Pr}H	500	39 000	2.1	[36]
IrH_2_(POCOP)^[c]^	333	55 000	2.9	[1, 10, 55]
[FeCp(CO)_2_]_2_/*hν*	20	64 000	1.8	[31]
RuH_2_(PMe_3_){NMe((CH_2_)_2_P*i*Pr_2_)_2_}	333	191 000	1.8	[27]
H_2_BN*i*Pr_2_+NMeH_2_ (stoichiometric)	–	20 000 [*M*_w_ 201 000]	10.2	[48]
[MeH_2_N⋅BH_2_(OEt_2_)][OTf]+base	–	*M*_w_<5000	–	[14]

[a] Calibrated relative to polystyrene standards; [b] Data in parentheses using H_2_ as a chain termination agent; [c] Representative *M_n_* values reported.

1. Low molecular weight: *M_n_*<5000 g mol^−1^
2. Medium molecular weight: *M_n_* 5000–50 000 g mol^−1^
3. High molecular weight: *M_n_* 50 000–200 000 g mol^−1^
4. Very high molecular weight: *M_n_*>200 000 g mol^−1^

**Scheme 3:**
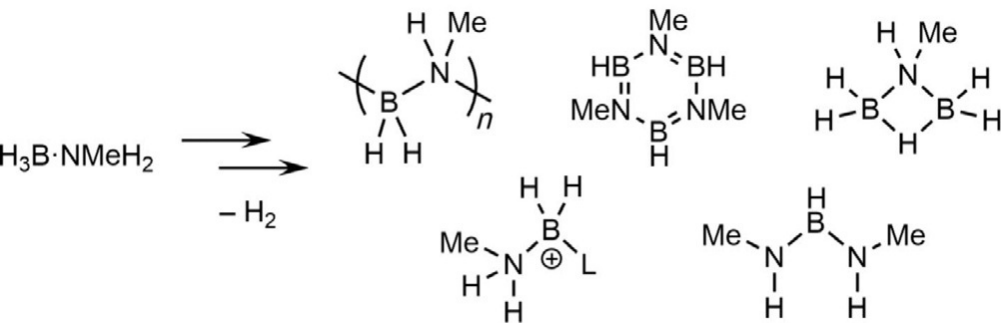
Commonly observed products from H_3_B⋅NMeH_2_ dehydropolymerization (L=NMeH_2_, coordinating solvent).

Selectivity is also a key factor to consider when addressing catalyst performance. Catalysts which selectively release one equivalent of hydrogen and form high molecular weight polymer are desirable, rather than borazine/polyborazylene formation and other side products. Release of more than one equivalent of hydrogen produces borazine (HBNR)_3_ and polyborazylene (BNH)_*x*_ products in the case of H_3_B⋅NH_3_.^56^ B−N cleavage is often also observed, such as BH(NRH)_2_, H_2_B(μ‐H)(μ‐NRH)BH_2_ and [BH_2_(NRH_2_)_2_]^+^ (Scheme 3), which reduce the yield and compositional purity of polymer.^28^, ^36^, ^57^, ^58^

## 3 Mechanism

Although a wide range of catalysts have been reported for the dehydropolymerization of amine–boranes mechanistic understanding is generally rather limited, in part due to the high degree of complexity associated with studying multiple processes occurring contemporaneously: dehydrogenation, polymerization and the production of off‐cycle products such as borazines and B−N cleavage products. Here we will consider some key experimental and theoretical findings that act as way markers in revealing the overall mechanism(s) of dehydropolymerization.

### 3.1 Dehydrogenation

**Scheme 4:**
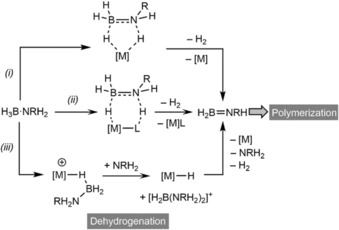
Generic mechanisms of dehydrogenation. (i) Inner sphere BH/NH activation, (ii) ligand assisted cooperative mechanism or (iii) hydride abstraction/boronium co‐catalysis. Dotted lines do not indicate the order of bond breaking/forming events.

Mechanistic studies of the dehydrogenation of amine–boranes are technically and theoretically simpler to study than overall dehydropolymerization, and this process has been studied in detail for a number of systems, often including model systems such as H_3_B⋅NMe_2_H that form simple dimers, for example, (H_2_BNMe_2_)_2_, rather than macromolecules. Typically, this is performed experimentally by measuring the volume or pressure of H_2_ gas liberated. As well as determining the number of H_2_ released per monomer unit [for example, one for polyaminoborane: *n* H_3_B⋅NR′H_2_→(H_2_BNR′H)_*n*_+*n* H_2_] temporal profiles provide kinetic information, and resulting kinetic isotope effects using deuterated amine–boranes^35^, ^36^, ^54^ help establish the rate‐ or turnover‐determining step, all of which can be supported by computational studies.^6^ In overall catalytic dehydropolymerization, the initial dehydrogenation is a metal‐promoted process and can potentially proceed by one of three established routes: (i) inner sphere BH/NH activation, (ii) ligand assisted cooperative mechanisms or (iii) hydride abstraction/boronium co‐catalysis (Scheme 4). The formation of σ‐amine–borane complexes, which have three‐center‐two‐electron M⋅⋅⋅H−B interactions,^59^ is central to many of these mechanisms.

For scenarios (i) and (ii) B−H and N−H activation can proceed stepwise in either order, with N−H activation^35^, ^36^ often (although not exclusively) the turnover limiting step, or in a concerted manner.^60^, ^61^ Mechanism (iii) operates by hydride abstraction at a cationic metal center to give a neutral metal‐hydride and a boronium cation [H_2_B(NRH_2_)(L)]^+^ (L=solvent, NRH_2_ from B−N bond cleavage), which is subsequently deprotonated by the same metal hydride to yield the aminoborane and recover the cationic metal center.^16^, ^36^, ^62^

Turnover numbers (ToN) and turnover frequencies (ToF) can be used to benchmark catalyst performance for dehydrogenation. However, comparisons between catalysts so far reported are not straightforward as ToFs have been calculated at one equivalent of H_2_ release (ToF),^36^ at 50 % conversion (ToF_50 %_),^38^ or at the maximum rate (ToF_max_),^35^ and hence cannot be directly compared. Moreover, as ToF values are generally concentration dependent they should be, ideally, measured under the same conditions using multiple data points.^63^ These general comments aside, ToF_max_ of 20 s^−1^ (72 000 h^−1^) has been reported for H_3_B⋅NH_3_ dehydrogenation by RuH(PMe_3_){N((CH_2_)_2_P*i*Pr_2_)_2_},^35^ whereas for H_3_B⋅NMeH_2_ dehydrogenation a range of ToFs have been reported, for example, 2400 h^−1^ for IrH_2_(POCOP),^27^ 1500 h^−1^ for Rh(Xantphos‐*i*Pr)H^36^ and 250 h^−1^ for [Rh{Xantphos‐Ph}{H_2_BNMe_3_(CH_2_)_2_ *t*Bu}][BAr^F^_4_] (Ar^F^=3,5‐C_6_H_3_(CF_3_)_2_).^37^ ToNs of up to 10 000 have been measured for H_3_B⋅NH_3_ dehydropolymerization,^35^ but values around 20–500 are typically reported for H_3_B⋅NMeH_2_ dehydropolymerization. This can be increased by recharging the catalyst system, which has been demonstrated for up to three cycles (ToN up to 1500).^27^, ^36^ Schneider has reported threefold improvements in ToN for H_3_B⋅NH_3_ dehydropolymerization using Fe and Ru pincer systems upon addition of a catalytic amount of NMe_2_Et.^32^ The added amine prevents catalyst deactivation by BH_3_ binding, produced from the rearrangement of 2 H_2_B=NH_2_ to BH_3_⋅THF and HB(NH_2_)_2_.^64^

### 3.2 Polymerization

**Figure 2:**
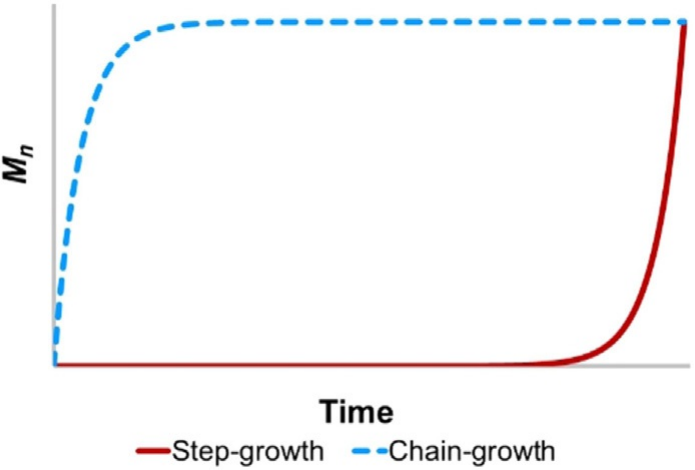
Polymer growth kinetic profiles for conventional chain‐growth and step‐growth polymerization mechanisms.

Although controlled dehydrogenation occurs by a metal‐centered process, polymerization can, in principle, proceed either on‐ or off‐metal. Current data is limited to a relatively small number of examples that have been studied in detail. For many of these, polymer growth kinetics reveal propagation by B−N forming chain‐growth mechanisms. Classical chain‐growth is characterized by significant molecular weight polymer being observed at early conversions and gradual consumption of monomer, whereas classical step‐growth only shows higher molecular weight polymer being formed at very high conversions, monomer is consumed early and the first products formed are short oligomers, Figure 2.^65^

**Scheme 5:**
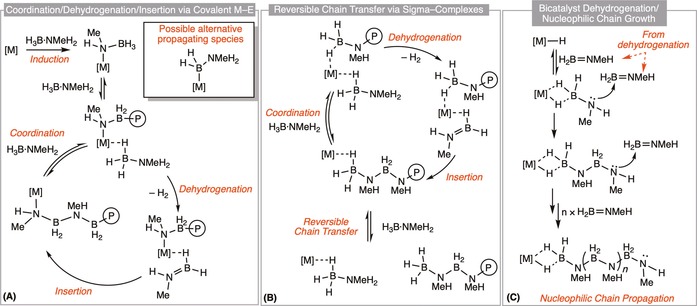
Principle suggested mechanistic pathways for amine–borane dehydropolymerization (P in the circle refers to polymer). Only selected, key, intermediates are shown and some of the steps represent telescoped elementary processes.

In general the mechanism‐led design of polymerization necessarily requires the elementary processes of initiation, propagation and termination steps to be understood and controlled. For amine–borane dehydropolymerization the propagating aminoborane monomer units are unstable with respect to oligomerization, making first‐formed species difficult to study. Initiation has thus not been considered in detail although examples of simple aminoboranes interacting with metal centers have been reported.^16^, ^17^, ^52^, ^66^, ^67^ Most questions that have been posed relate to chain propagation, or B−N coupling events, for which a number of mechanistic regimes have been proposed: coordination/dehydrogenation/insertion chain‐growth via a covalent M−E unit, dehydrogenation/insertion via sigma‐bound complexes that undergo reversible chain‐transfer and bicatalyst dehydrogenation/nucleophilic chain‐growth (Scheme 5).

The cationic rhodium bis(arylphosphine) system [Rh(Xantphos‐Ph){H_2_BNMe_3_(CH_2_)_2_ *t*Bu}][BAr^F^_4_] shows behavior consistent with a single‐site coordination/dehydrogenation/insertion mechanism (Scheme 5 A).^37^ The mechanism suggested proceeds by on‐metal dehydrogenation to give H_2_B=NMeH coordinated to the metal, which inserts into the growing polymer chain at the metal center, postulated to be anchored by a Rh‐amido linkage, although Rh−B linkages are also possible.^23^

**Scheme 6:**
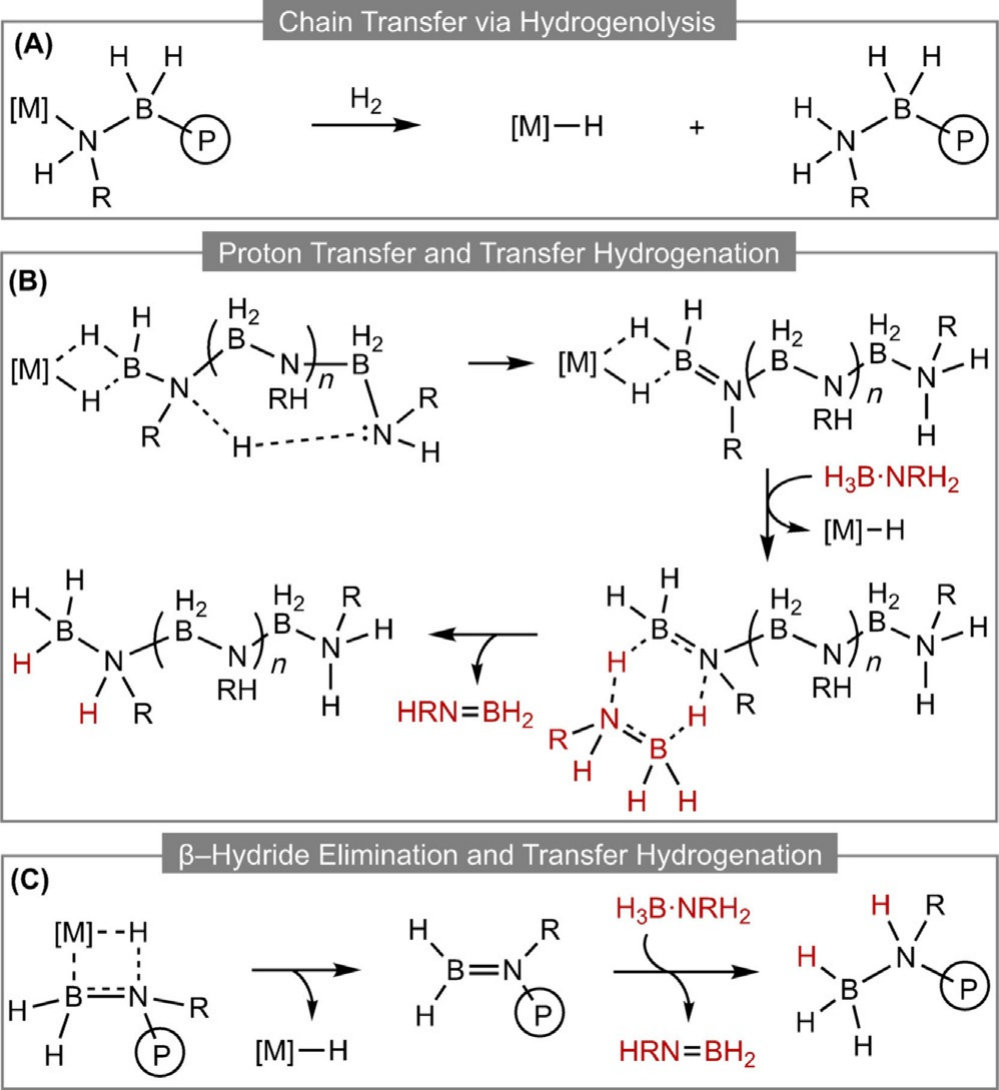
Suggested mechanistic pathways for termination in amine–borane dehydropolymerization (P in the circle refers to polymer).

This mechanism is proposed to be characterized by a decrease in polymer molecular weight with increased catalyst loading (as there are more active propagating sites per monomer unit) and a sensitivity to H_2_, which is proposed to act as a chain‐modifying agent by hydrogenolysis of the Rh−N (or Rh−B) bond (Scheme 6). On‐metal oligomerization by a mechanism which involves more weakly bound sigma‐complexes and reversible chain transfer has been proposed for the [IrH_2_(H_2_)_2_(PCy_3_)_2_][BAr^F^_4_] system (Scheme 5 B). These studies also highlighted the importance of additional amine–borane or aminoborane in promoting dehydrogenation through dihydrogen interactions.^69^, ^70^

Paul has suggested, using DFT calculations, a mechanism for dehydropolymerization catalyzed by IrH_2_(POCOP), which resembles a nucleophilic head‐to‐tail polymerization (Scheme 5 C).^71^ Separate metal centers (i.e., “bicatalyst”) are responsible for the dehydrogenation^61^ and polymerization activity. This latter process involves metal‐initiated chain‐growth in which the metal binds one end of the polymer chain, and aminoborane monomer units are added by nucleophilic attack of the amine terminus lone pair. This type of mechanism is proposed for systems which display modified chain‐growth behavior,^27^, ^36^ in which there is no clear correlation between molecular weight and catalyst loading, on account of the dual role of the metal centers in dehydrogenation and polymerization processes, which makes it difficult to resolve the catalyst contribution to polymer growth kinetics due to potential complications stemming from the presence of multiple, competing equilibria. This mechanism is similar to that proposed by Schneider for RuH_2_(PMe_3_){NH((CH_2_)_2_P*i*Pr_2_)_2_}, in which different Ru sites catalyze dehydrogenation and B−N coupling, reliant on cooperative assistance from the PNP ligand.^35^ As such, the mechanism does not involve an anchoring M−N (or M−B) bond for chain propagation, and it is also proposed that H_2_ does not act to modify the polymer chain length. An unusual mechanistic pathway calculated by Rossin and Shubina involves B−N cleavage chain‐growth, in which BH_3_ and NH_3_ moieties are added individually at a cobalt tetraphosphine center.^53^

These three proposed mechanisms involve the generation of aminoborane, either free or bound to a metal center. Baker proposed an elegant probe for these two extremes using entrapment of aminoborane by hydroboration of exogenous cyclohexene.^68^ There are caveats associated with this approach though, as the trapping is dependent on the relative rates of hydroboration versus polymerization, where nucleophilic assistance of the solvent can be key.^64^ Interestingly Paul has calculated B−N head‐to‐tail bond forming polymerization using the IrH_2_(POCOP) catalyst to be lower in energy than cyclohexene hydroboration;^6^, ^71^ suggesting that even if free aminoborane is formed in this specific system, polymerization could well be favored over hydroboration in the experimental system.

In the context of amine–borane dehydropolymerization, very little is known about chain‐transfer/termination events (Scheme 6). As mentioned, in some systems hydrogen has been found to act as a chain‐modifying agent, which causes additional complexity as H_2_ is a product of dehydropolymerization.^37^, ^54^ Such modification is proposed to operate during a coordination/dehydrogenation/insertion chain‐growth mechanism (Scheme 5 A), where hydrogenolysis of a Rh−N or Rh−B bond by H_2_ leads to termination of the growing chain (Scheme 6 A). This has direct links to control of polymer molecular weight in olefin polymerization.^72^ Mechanisms that do not involve a covalent M−E bond would be expected to be less sensitive to H_2_ pressure. For example, Paul has calculated the chain termination process in IrH_2_(POCOP) catalyzed dehydropolymerization of H_3_B⋅NH_3_, Scheme 5 C,^71^ where chain termination is proposed to occur by proton transfer to the propagating amine unit, dissociation of the Ir center and transfer hydrogenation^15^, ^73^ of the H_2_B=NH‐polymer end group by free H_3_B⋅NH_3_ (Scheme 6 B). Experimentally the system has been shown to be insensitive to H_2_ pressure,^27^ as have others^33^, ^36^ where there is, interestingly, no clear correlation between catalyst loading and degree of polymerization.

An alternative chain termination process involves β‐hydride elimination from a M–amido‐ or M–boryl‐anchored polymer, possibly coupled with transfer hydrogenation of the aminoborane end group (Scheme 6 C). Such processes have been modelled in related phosphine–borane model systems.^23^ In principle, such a β‐hydride transfer pathway could be probed by deuterium labelling of the substrate, as reported recently for N‐methylamine–borane.^54^ Although no strong trend was observed, polymers with N−D substituents yielded slightly higher molecular weight material, as measured by GPC, which could indicate that N−D cleavage is the rate‐determining step of termination. End group analysis would provide valuable information about termination processes, and definitive studies in this area would be invaluable for understanding these events. Living polymerization systems would obviously aid this analysis.

**Figure 3:**
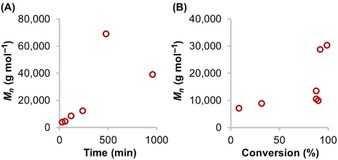
**(A)** *M_n_* of (H_2_BNMeH)_*n*_ produced after varying reaction times using TiCl_2_Cp*_2_/2×*n*BuLi showing step‐growth polymerization and subsequent depolymerization. **(B)** Polymer growth kinetics of hybrid chain‐growth/step‐growth dehydropolymerization using [Rh{Ph_2_P(CH_2_)_3_PPh_2_}(η^6^‐C_6_H_5_F)][BAr^F^_4_]. Adapted from Ref. 28, 54, respectively.

So far, the mechanisms discussed have incorporated the essential characteristics of chain‐growth. There are examples where an alternative step‐growth polymerization mechanism is proposed. For Manners’ TiCl_2_Cp^R^_2_/2×*n*BuLi catalyst system,^28^ high molecular weight polymer is only observed at high conversions (i.e., after 8 hours, Figure 3 A), monomer is consumed early on, and increased catalyst loading produces higher degrees of polymerization. In contrast, Weller's [Rh{Ph_2_P(CH_2_)_3_PPh_2_}(η^6^‐C_6_H_5_F)][BAr^F^_4_] catalyst^54^ displays an unusual hybrid chain‐growth/step‐growth mechanism (Figure 3 B). Relatively long chain polymers were produced very early on in the reaction, polymer molecular weights decreased with higher catalyst loadings, monomer was only consumed gradually and the system is sensitive to hydrogen, signaling a coordination/insertion/chain‐growth process for the first ca 90 % of reaction. This is amplified by a late‐stage step‐growth process that rapidly increases the molecular weight of the polymer. In both systems, further support for a step‐growth mechanism was obtained by subjecting isolated low molecular weight polymer to catalyst, which produced higher molecular weight material indicative of coupling of the polymer chains.

**Figure 4:**
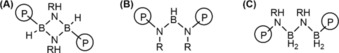
Possible step‐growth coupling motifs (P in the circle refers to polymer).

Possible step‐growth coupling motifs have been proposed for the Rh‐system, as shown in Figure 4. ^11^B NMR data point towards a linear head‐to‐tail coupling as the most likely linkage, that is, Figure 4 C.^54^ As with chain‐growth processes, model system designed to probe step‐growth coupling processes in amine–borane dehydropolymerization would be beneficial.

### 3.3 Depolymerization

**Scheme 7:**
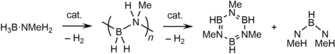
Dehydropolymerization of H_3_B⋅NMeH_2_ and subsequent depolymerization of (H_2_BNMeH)_*n*_ by TiCl_2_Cp^R^_2_/2×*n*BuLi.

Catalyzed depolymerization of the formed polyaminoborane has been shown to occur. In the TiCl_2_Cp^R^_2_/2×*n*BuLi catalyzed synthesis of (H_2_BNMeH)_*n*_, a decrease in *M_n_* was observed when extended reaction times were used (16 hours), Figure 3 A,^28^ which was attributed to depolymerization of (H_2_BNMeH)_*n*_, to eventually form (HBNMe)_3_ and HB(NMeH)_2_ (Scheme 7). Similar polymer depolymerization has been described with the [FeCp(CO)_2_]_2_/*hν* system, wherein (H_2_BNMeH)_*n*_ was converted to (HBNMe)_3_ over 13 hours.^31^ The mechanism of depolymerization is unresolved, but may bear similarity to observations in model linear diborazane systems, where IrH_2_(POCOP) catalyzed redistribution of H_3_BNMeHBH_2_NMe_3_ gave a mixture of species, (H_2_BNMeH)_*n*_ (*M_n_*<5000 g mol^−1^), H_3_B⋅NMe_3_, H_3_B⋅NMeH_2_ and (HBNMe)_3_.^58^ Clearly, if the same catalyst system mediates both polymerization and deleterious depolymerization then careful studies are required to find the optimum conditions for polymer isolation, as well as removal of any residual catalyst from the polymer to stop unwanted further reaction. Targeted depolymerization of (H_2_BNMeH)_*n*_ has been demonstrated by Manners, by addition of one equivalent per repeat unit of a strong Lewis‐base (i.e., an N‐heterocyclic carbene, NHC) to give the corresponding aminoborane‐NHC adduct.^55^

### 3.4 Residual catalyst in polyaminoborane

Recently, the amount of residual catalyst that remains in the polymer after precipitation from solution has been quantified using NMR and ICP‐MS techniques. For example, using [Rh(Xantphos‐*i*Pr)][BAr^F^_4_]‐based systems, even low catalyst loadings (0.2 mol %) resulted in 450 ppm (i.e., 0.045 mol %) of Rh‐content in the polymer.^36^ The [BAr^F^_4_]^−^ anion was also associated with the polymer, which is problematic because even very low levels show a strong response in the resulting GPC analysis, using refractive index detection, that must be accounted for. Similar relative levels of residual metal have been noted in phosphine–borane dehydropolymerization using Fe‐based catalysts.^74^ Measuring and then removing, or at the very least deactivating, residual catalyst would be very useful with regard to longer term stability and processing of polymeric materials, especially as metal catalyzed hydrolysis^75^ of polyaminoboranes is a possible decomposition route. In this regard the recently reported catalyst‐free routes may offer significant advantages.^48^

## 4 Substrate Scope

**Figure 5:**
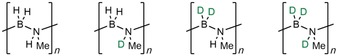
Deuterated polyaminoborane isotopologues.

The outcome of dehydropolymerization is also dependent on the steric and electronic properties of the substituents on the amine–borane. The current state‐of‐the art indicates that polymer formation appears to require sterically undemanding substituents at either B‐ or, more commonly, N‐ and as such is only currently known for primary amines or boranes. Dehydrogenation of secondary amines and boranes, or bulky primary examples (e.g., H_3_B⋅N*t*BuH_2_) yields oligomers, monomeric aminoboranes (e.g., H_2_B=N*i*Pr_2_) or cyclic borazanes [e.g., (H_2_BNMe_2_)_2_]. Deuterated polyaminoboranes have been recently prepared from H_3_B⋅NMeD_2_, D_3_B⋅NMeH_2_ and D_3_B⋅NMeD_2_ to form the isotopologues (R_2_BNMeR′)_*n*_ (R, R′=H or D, Figure 5) that were found to possess physical properties similar to (H_2_BNMeH)_*n*_.^54^ Interestingly, the same catalyst that was used for dehydropolymerization, [Rh{Ph_2_P(CH_2_)_3_PPh_2_}(η^6^‐C_6_H_5_F)][BAr^F^_4_], was also used to prepare the deuterium‐labeled on boron precursor amine–boranes by H/D exchange with D_2_.

### 4.1 N‐substitution

**Figure 6:**
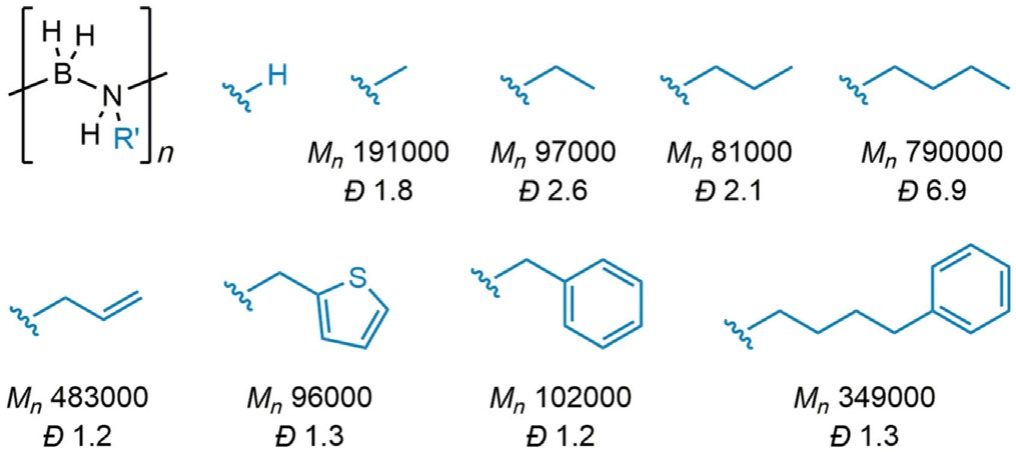
Currently reported N‐substituted polyaminoboranes (*M_n_*=g mol^−1^). *M_n_* and *Ð* values for (H_2_BNH_2_)_*n*_ have not been measured due to the insolubility of the material.

The most widely used substrate in dehydropolymerization studies is H_3_B⋅NMeH_2_, rather than the parent ammonia–borane H_3_B⋅NH_3_. The advantage of improved solubility, particularly of the polymer produced, tends to outweigh the convenient commercial availability of H_3_B⋅NH_3_ as a substrate. A variety of other N‐alkyl‐substituted polyaminoboranes have now been reported (H_2_BNRH)_*n*_, R=H, Me, Et, *n*Pr, *n*Bu, CH_2_Ph, (CH_2_)_4_Ph, CH_2_(C_4_H_3_S) and CH_2_CH=CH_2_ (Figure 6), through catalytic routes from the appropriate primary amine–boranes, or stoichiometric BH_2_ transfer routes.^1^, ^28^, ^48^, ^76^ N‐aryl‐substituted amine–boranes H_3_B⋅NRH_2_ (R=Ph, *p*‐C_6_H_4_CF_3_, *p*‐C_6_H_4_OMe) undergo spontaneous dehydrocoupling at room temperature to an array of products, hampering their use as monomers.^77^ Here, future low temperature studies on catalysis may be beneficial.

### 4.2 B‐substitution

**Figure 7:**
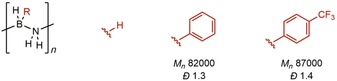
Known B‐substituted polyaminoboranes (*M_n_*=g mol^−1^). *M_n_* and *Ð* values for (H_2_BNH_2_)_*n*_ have not been measured due to the insolubility of the material.

Polyaminoboranes bearing non‐hydrogen substituents at boron are more limited. Attempts to prepare B‐methylated polymers gave intermediate species which were tentatively assigned to (HMeBNH_2_)_*n*_ oligomers and polymers. These were unstable towards further dehydrogenation, yielding borazine (MeBNH)_3_ and bis(amino)borane MeB(NH_2_)_2_.^78^ Rationalizing that the increased electron density at boron weakens the B−N bond, Manners has successfully incorporated aryl groups for the preparation of (HRBNH_2_)_*n*_ (R=Ph, *p*‐C_6_H_4_CF_3_) polymers, which represent the first BN‐backbone analogues of polystyrene (Figure 7).^79^ Interestingly, the *p*‐CF_3_ B‐substituted polyaminoboranes show much improved thermal stability over the parent (HPhBNH_2_)_*n*_, attributed to the electron‐withdrawing ability of the CF_3_ substituent. Dehydropolymerization of B‐ and N‐substituted amine–boranes to produce stable polymers (HRBNR′H)_*n*_ has yet to be realized, although (HMeBNMeH)_*n*_ has been transiently observed.^78^

### 4.3 Copolymers

Random copolymers have been prepared from the copolymerization of two amine–borane monomers, which have provided access to high molecular weight materials. An attraction of this approach is the modified solubility of the product. (H_2_BNH_2_)_*n*_ is insoluble, whereas (H_2_BNH_2_)_*n*_‐*r*‐(H_2_BNMeH)_*m*_ (*n*:*m*=1:1, 1:3) copolymers are soluble.^1^, ^27^ (H_2_BNMeH)_*n*_‐*r*‐(H_2_BN*n*BuH)_*m*_ (*n*:*m*=1:1, 1:3), and {H_2_BN(CH_2_Ph)H}_*n*_‐*r*‐(H_2_BN*n*BuH)_*m*_ (*n*:*m*=2:1) copolymers have been similarly prepared using IrH_2_(POCOP)^1^ and TiCl_2_Cp*_2_/2×*n*BuLi^28^ catalyzed dehydropolymerization, respectively. Alcaraz has shown that this BH_2_ transfer methodology is similarly amenable to copolymerization, yielding (H_2_BNEtH)_*n*_‐*r*‐(H_2_BNRH)_*m*_ (R=*n*Bu, *n*:*m*=1:1; R=*n*Pr, *n*:*m*=1:2).^48^ The cross‐linker hydrazine–diborane has also been used in copolymerization with H_3_B⋅NH_3_, which offered an improved ceramic yield over (H_2_BNH_2_)_*n*_.^1^ This could be important when exploring their utility as precursors to BN materials.

## 5 Polymer Characterization

There are a number of methods used to routinely characterize polyaminoboranes, including NMR spectroscopy, GPC and mass spectrometry, as detailed next. Identification of B−H and N−H stretching modes by infrared spectroscopy has also been used.^1^

### 5.1 NMR spectroscopy

^11^B NMR spectroscopy offers a straightforward characterization technique for polyaminoboranes. The quaternary B centers in H_3_B⋅NRH_2_ precursors display broad (due to quadrupolar coupling) quartet resonances [for example, *δ*(^11^B) −18.8 (^1^ *J*_BH_ 94 Hz); R=Me, CDCl_3_)]^80^ which shift downfield in the (H_2_BNRH)_*n*_ polymer [for example, *δ*(^11^B) −6.7; R=Me, CDCl_3_]^1^ where they are typically observed as broad resonances without resolvable ^1^H coupling, except in the case of low molecular weight polymer or oligomeric species.^70^ Solid state and solution ^11^B NMR spectroscopy has been used to propose chain branching/cross‐linking at the boron center, signaled by a resonance to lower field of the main polymer signal.^35^, ^36^, ^38^, ^81^ ^1^H and ^13^C NMR spectroscopy provide information about the R group environments, and have been used to make inferences regarding polymer tacticity, although no clear correlations have yet been developed.^1^, ^27^ ^15^N NMR spectroscopy of polyaminoboranes has not been studied in detail, but Beweries has reported the ^1^H‐^15^N HMQC NMR spectrum of (H_2_BNMeH)_*n*_, which displays a cross‐peak at *δ*(^15^N) −365 with *J*_NH_≈65 Hz.^33^ Solid‐state ^11^B NMR can also give information about B‐environments.^27^, ^35^, ^50^ Despite these observations, as there are only a few oligomeric systems synthesized that provide models for chain branching and different polymer chain stereochemistries, correlations between NMR chemical shifts and chain structure remain to be developed.

Quantitative end group analysis by NMR spectroscopy has yet to be described, perhaps as a consequence of the termination event in polymerization not being well defined. Such analysis is complicated by the fact that polymer samples have a tendency to incorporate small quantities of unreacted amine–borane which can mask end group signals (i.e., ‐BH_3_). This aside, there is the attractive possibility of comparing the relative integral of an BH_3_ end‐group with the main chain B‐environments and correlating with GPC data, as qualitatively described recently by Manners.^28^

### 5.2 Mass spectrometry

**Figure 8:**
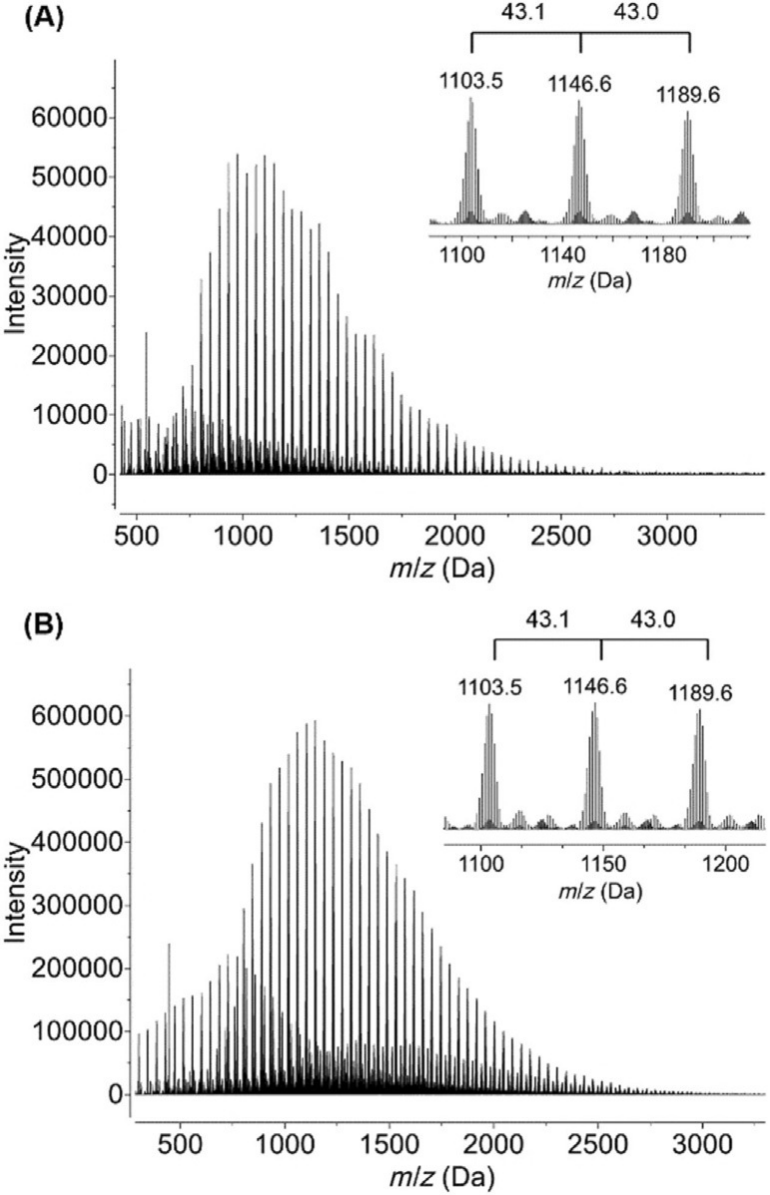
**(A)** ESI‐MS(+) of (H_2_BNMeH)_*n*_ prepared using IrH_2_(POCOP) (*M_n_* 107 000 g mol^−1^, *Ð* 2.0). **(B)** ESI‐MS(+) of (H_2_BNMeH)_*n*_ prepared using [Rh{Ph_2_P(CH_2_)_3_PPh_2_}(η^6^‐C_6_H_5_F)][BAr^F^_4_] (*M_n_* 28 000 g mol^−1^, *Ð* 1.8).

Mass spectrometry methods have been utilized for the characterization of polyaminoboranes, mainly Electrospray ionization mass spectrometry (ESI‐MS).^27^, ^36^, ^82^ Polyaminoboranes exhibit multiple distributions in ESI‐MS (in both positive and negative ionization modes), but the degree of polymerization observed is always significantly lower than that determined by GPC, a clear distinction between the techniques. It is also levelled to be rather similar irrespective of the degree of polymerization determined by GPC, for example, Figure 8, suggesting that it is a technique that is rather insensitive to polymer chain length, and is best deployed to interrogate the repeat units only.

### 5.3 Gel permeation chromatography

Measurement of polymer molecular weight and dispersity is achieved by gel permeation chromatography (GPC, or size exclusion chromatography), where molecular weights of up to *M*_w_ 5 450 000 g mol^−1^ have been measured for (H_2_BN*n*BuH)_*n*_.^48^ This technique relies on the hydrodynamic radius of the polymer in solution (generally run in 0.1 % w/w [N*n*Bu_4_]Br in THF solutions to reduce tailing due to column adsorption effects),^27^ calibrated relative to polystyrene standards and often using Refractive Index detection. Little is known about the nature of polyaminoboranes in solution, although dihydrogen interactions have been shown to be important in H_3_B(NH_2_BH_2_)_*n*_NH_3_ oligomers.^83^, ^84^ Light‐scattering experiments conducted by Manners suggest that GPC may overestimate (H_2_BNMeH)_*n*_ molecular weight by a factor of 3 to 6,^27^ and crucial to the area is that such calibrations are explored further and in more detail.^85^

### 5.4 Materials properties characterization and application

Studies on the material properties of polyaminoboranes have been more limited. Wide‐angle X‐ray scattering (WAXS) of (H_2_BNRH)_*n*_ produced using IrH_2_(POCOP) found the material was semicrystalline in the case of R=H but amorphous when R=Me.^1^, ^27^ Thermogravimetric analysis of polyaminoboranes generally demonstrates no clear link between decomposition temperature or ceramic yield and polymer molecular weight or substitution pattern,^1^, ^27^, ^48^, ^54^ and the effects of polymer microstructure on ceramic and material properties still need to be delineated.

**Figure 9:**
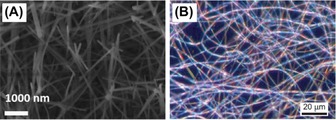
**(A)** SEM image of Al_5_BO_9_ nanowires grown by pyrolysis of (H_2_BNH_2_)_*n*_ on sapphire wafer.^10^ Copyright 2016, The Authors. Published by The Royal Society of Chemistry. **(B)** Optical light microscope image of electrospun fibers of (H_2_BNMeH)_*n*_. Reproduced with permission from Ref. 3. Copyright 2013, Nature Publishing Group.

Pyrolysis of (H_2_BNH_2_)_*n*_ gave a semicrystalline material which displayed a broad N−H absorption in the IR spectrum but no B−H stretch, and two major peaks by powder X‐ray diffraction, one of which corresponds to boron nitride.^1^ Indeed, oligomeric H_3_B(NH_2_BH_2_)_*n*_NH_3_ (*n*=1, 2) has been used as a chemical vapor deposition precursor to hexagonal boron nitride (h‐BN).^11^ Pyrolysis of polyaminoboranes (H_2_BNRH)_*n*_ (R=H, Me) on a range of substrates led to formation of a variety of materials, including porous h‐BN, amorphous B and N containing nanostructures and spherical nanoparticles.^10^ Pyrolysis on sapphire wafers, Al_2_O_3_ or AlN powders gave crystalline Al_5_BO_9_ nanowires, nanotubes and nanoribbons, the diameter of which could be tuned by surface defect density (Figure 9 A). (H_2_BNMeH)_*n*_ can be electrospun to produce fibers (Figure 9 B).^3^ Much more work is needed with regard to both producing and studying materials based on polyaminoboranes in order to capitalize on the opportunities these materials offer.

## 6 Challenges and Opportunities

Throughout this Concept article we have attempted to highlight the current state of the art in amine–borane dehydropolymerization. Over the last ten years there is no doubt that significant progress has been made in the development of new catalysts, mechanistic‐understanding and substrate scope. Nevertheless, challenges remain that need to be addressed if those working in, or coming new to, the field want to transition it from a largely academic exercise to one where new functional materials are made to order. We have outlined these throughout the article and bring them together to suggest four overarching challenges, and the opportunities their resolution will offer.

**1) Mechanism**: A more complete understanding of the underlying mechanistic schemes that operate for dehydropolymerization, and the desirable benefits that each offer in terms of polymeric material they produce, is needed. Initiation and termination events are particularly poorly understood, and control of these may allow for living polymerization—and thus block copolymers—to be produced. The development of controlled polymerization using non‐catalyzed routes that potentially offer wider substrate scope and no active catalyst entrapment are likely to also be important.

**2) Performance criteria**: A set of common performance criteria that are deployed for polymer synthesis is needed, that will allow the community to calibrate and reproduce experiments. We suggest that the particular performance parameters (as measured by the polymer and materials characteristics determined—see points 3 and 4) covers optimizing the following parameters: catalyst:substrate ratio where appropriate, systems open or closed to H_2_ buildup, temperature (especially lower temperatures), solvent, temporal profiles (to probe for depolymerization and mechanism) and active catalyst in polymer. Reaction kinetics present a particular problem for standardization, as turnover frequencies are, of course, concentration and temperature dependent.^60^ Until a suitable, readily accessible benchmark system is discovered we simply suggest using units of concentration as well as mol % to describe catalyst loading, and the routine inclusion of temporal profiles for dehydropolymerization as supporting materials.

It is important to state that the “best performing” polyaminoborane for any particular application is currently unknown. We thus suggest current efforts should be focused upon controlling polymer yield, structure and compositional purity as well as scalability of synthesis, rather than simply aiming for high‐molecular weight polymer with narrow dispersity, although this is obviously one set of desirable properties.

**3) Structure–activity relationships** are needed for a range of systems, that identify the key characteristics that influence or control degree of polymerization, dispersity, chain branching, stability (especially towards hydrolysis and increased temperature) and material properties. This encompasses both new catalyst design and monomers. An obvious target is the development of systems that induce stereocontrol in the main‐chain backbone so that tacticity of the resulting polymer can be controlled, mimicking that successfully deployed in polyolefin chemistry.^13^ Here new model oligomeric complexes with well‐defined stereochemistry and substitution patterns will be invaluable in providing spectroscopic handles for polymer characterization.

**4) Polymer and materials properties**: A natural, and necessary, extension of the development of a wide variety of new polymeric materials is their materials properties. Such studies will most likely need increased input from materials scientists and potential end users (specialty plastics, energy and new electronic‐ and coating‐materials communities in particular). Ultimately, we believe that such collaboration will help focus the community in delivering polyaminoboranes “to order” that find use in a potentially wide‐variety of applications.

The decade since the identification of metal catalyzed amine–borane dehydropolymerization has witnessed significant progress in the field, and it is likely that the next decade ahead will see many of the challenges outlined above overcome and the resulting opportunities for continued expansion of both the synthesis and use of polyaminoborane materials harnessed. We look forward to such developments, as well as welcoming more new researchers to this challenging, often frustrating but ultimately very rewarding research field.

## Conflict of interest

The authors declare no conflict of interest.

## References

[1] A. Staubitz , A. P. Soto , I. Manners , Angew. Chem. Int. Ed. 2008, 47, 6212–6215; 10.1002/anie.200801197 18613187

[2] V. Pons , R. T. Baker , Angew. Chem. Int. Ed. 2008, 47, 9600–9602; 10.1002/anie.200803089 19006157

[3] E. M. Leitao , T. Jurca , I. Manners , Nat. Chem. 2013, 5, 817–29. 24056337 10.1038/nchem.1749

[4] A. Staubitz , A. P. M. Robertson , M. E. Sloan , I. Manners , Chem. Rev. 2010, 110, 4023–4078. 20672859 10.1021/cr100105a

[5a] H. C. Johnson , T. N. Hooper , A. S. Weller in Synth. Appl. Organoboron Compd. (Eds.: E. Fernández, A. Whiting), Springer, Cham, 2015, pp. 153–220;

[5b] D. Han , F, Anke , M. Trose , T. Beweries , Coord. Chem. Rev. 2019, 380, 260–287.

[6] S. Bhunya , T. Malakar , G. Ganguly , A. Paul , ACS Catal. 2016, 6, 7907–7934.

[7] A. Rossin , M. Peruzzini , Chem. Rev. 2016, 116, 8848–8872. 27075435 10.1021/acs.chemrev.6b00043

[8] A. Staubitz , Angew. Chem. Int. Ed. 2018, 57, 5990–5992; 10.1002/anie.201801903 29717536

[9] S. Bernard , P. Miele , Materials 2014, 7, 7436–7459. 28788257 10.3390/ma7117436 PMC5512645

[10] V. A. Du , T. Jurca , G. R. Whittell , I. Manners , Dalton Trans. 2016, 45, 1055–1062. 26649782 10.1039/c5dt03324a

[11] X. Wang , T. N. Hooper , A. Kumar , I. K. Priest , Y. Sheng , T. O. M. Samuels , S. Wang , A. W. Robertson , M. Pacios , H. Bhaskaran , A. S. Weller , J. H. Warner , CrystEngComm 2017, 19, 285–294.

[12] More than 178 m tons of polyolefins were manufactured in 2015. D. W. Sauter , M. Taoufik , C. Boisson , Polymer 2017, 9, 185.

[13] J. F. Hartwig , Organotransition Metal Chemistry, University Science Books, Sausalito, 2010.

[14] O. J. Metters , A. M. Chapman , A. P. M. Robertson , C. H. Woodall , P. J. Gates , D. F. Wass , I. Manners , Chem. Commun. 2014, 50, 12146–12149. 10.1039/c4cc05145a 25177756

[15] H. C. Johnson , A. S. Weller , J. Organomet. Chem. 2012, 721–722, 17–22.

[16] A. Kumar , N. A. Beattie , S. D. Pike , S. A. Macgregor , A. S. Weller , Angew. Chem. Int. Ed. 2016, 55, 6651–6656; 10.1002/anie.201600898 PMC5074255 27100775

[17] G. Alcaraz , L. Vendier , E. Clot , S. Sabo-Etienne , Angew. Chem. Int. Ed. 2010, 49, 918–920; 10.1002/anie.200905970 20039239

[18] E. Y.-X. Chen , Chem. Rev. 2009, 109, 5157–5214. 19739636 10.1021/cr9000258

[19] J. Choi , A. H. R. MacArthur , M. Brookhart , A. S. Goldman , Chem. Rev. 2011, 111, 1761–1779. 21391566 10.1021/cr1003503

[20] S. Aldridge , A. J. Downs , C. Y. Tang , S. Parsons , M. C. Clarke , R. D. L. Johnstone , H. E. Robertson , D. W. H. Rankin , D. A. Wann , J. Am. Chem. Soc. 2009, 131, 2231–2243. 19170515 10.1021/ja807545p

[21] D. J. Grant , M. H. Matus , K. D. Anderson , D. M. Camaioni , S. R. Neufeldt , C. F. Lane , D. A. Dixon , J. Phys. Chem. A 2009, 113, 6121–6132. 19422181 10.1021/jp902196d

[22] Borazine (RBNR′)_3_, and the products of B−N cleavage, such as BH(NRH)_2_, H_2_B(μ-H)NRHBH_2_ and [BH_2_(NRH_2_)_2_]^+^ characterize unselective and poor catalyst systems.

[23] T. N. Hooper , A. S. Weller , N. A. Beattie , S. A. Macgregor , Chem. Sci. 2016, 7, 2414–2426. 29997783 10.1039/c5sc04150c PMC6003611

[24] A. Schäfer , T. Jurca , J. Turner , J. R. Vance , K. Lee , V. A. Du , M. F. Haddow , G. R. Whittell , I. Manners , Angew. Chem. Int. Ed. 2015, 54, 4836–4841; 10.1002/anie.201411957 25712707

[25] M. C. Denney , V. Pons , T. J. Hebden , D. M. Heinekey , K. I. Goldberg , J. Am. Chem. Soc. 2006, 128, 12048–12049. 16967937 10.1021/ja062419g

[26] B. L. Dietrich , K. I. Goldberg , D. M. Heinekey , T. Autrey , J. C. Linehan , Inorg. Chem. 2008, 47, 8583–8585. 18785732 10.1021/ic801161g

[27] A. Staubitz , M. E. Sloan , A. P. M. Robertson , A. Friedrich , S. Schneider , P. J. Gates , I. Manners , J. Schmedt auf der Guenne , J. Am. Chem. Soc. 2010, 132, 13332–13345. 20806956 10.1021/ja104607y

[28] T. Jurca , T. Dellermann , N. E. Stubbs , D. A. Resendiz-Lara , G. R. Whittell , I. Manners , Chem. Sci. 2018, 9, 3360–3366. 29780466 10.1039/c7sc05395a PMC5933219

[29] T. Kakizawa , Y. Kawano , K. Naganeyama , M. Shimoi , Chem. Lett. 2011, 40, 171–173.

[30] R. T. Baker , J. C. Gordon , C. W. Hamilton , N. J. Henson , P. H. Lin , S. Maguire , M. Murugesu , B. L. Scott , N. C. Smythe , J. Am. Chem. Soc. 2012, 134, 5598–5609. 22428955 10.1021/ja210542r

[31] J. R. Vance , A. P. M. Robertson , K. Lee , I. Manners , Chem. Eur. J. 2011, 17, 4099–4103. 21387434 10.1002/chem.201003397

[32] A. Glüer , M. Förster , V. R. Celinski , J. Schmedt Auf Der Günne , M. C. Holthausen , S. Schneider , ACS Catal. 2015, 5, 7214–7217.

[33] F. Anke , D. Han , M. Klahn , A. Spannenberg , T. Beweries , Dalton Trans. 2017, 46, 6843–6847. 28504796 10.1039/c7dt01487b

[34] M. Käß , A. Friedrich , M. Drees , S. Schneider , Angew. Chem. Int. Ed. 2009, 48, 905–907; 10.1002/anie.200805108 19116993

[35] A. N. Marziale , A. Friedrich , I. Klopsch , M. Drees , V. R. Celinski , J. Schmedt auf der Günne , S. Schneider , J. Am. Chem. Soc. 2013, 135, 13342–13355. 23930890 10.1021/ja311092c

[36] G. M. Adams , A. L. Colebatch , J. T. Skornia , A. I. McKay , H. C. Johnson , G. C. Lloyd-Jones , S. A. Macgregor , N. A. Beattie , A. S. Weller , J. Am. Chem. Soc. 2018, 140, 1481–1495. 29286647 10.1021/jacs.7b11975

[37] H. C. Johnson , E. M. Leitao , G. R. Whittell , I. Manners , G. C. Lloyd-Jones , A. S. Weller , J. Am. Chem. Soc. 2014, 136, 9078–9093. 24844130 10.1021/ja503335g

[38] M. A. Esteruelas , P. Nolis , M. Oliván , E. Oñate , A. Vallribera , A. Vélez , Inorg. Chem. 2016, 55, 7176–7181. 27367792 10.1021/acs.inorgchem.6b01216

[39] P. Bellham , M. S. Hill , G. Kociok-Köhn , Dalton Trans. 2010, 44, 7587–7589. 10.1039/c5dt00178a 25789721

[40] J. Spielmann , D. F. J. Piesik , S. Harder , Chem. Eur. J. 2010, 16, 8307–8318. 20564288 10.1002/chem.201000028

[41] R. J. Less , R. L. Melen , D. S. Wright , RSC Adv. 2012, 2, 2191–2199.

[42] C. Appelt , J. C. Slootweg , K. Lammertsma , W. Uhl , Angew. Chem. Int. Ed. 2013, 52, 4256–4259; 10.1002/anie.201208746 23471587

[43] Z. Mo , A. Rit , J. Campos , E. L. Kolychev , S. Aldridge , J. Am. Chem. Soc. 2016, 138, 3306–3309. 26918906 10.1021/jacs.6b01170

[44] M. Boudjelel , E. D. S. Carrizo , S. Mallet-Ladeira , S. Massou , K. Miqueu , G. Bouhadir , D. Bourissou , ACS Catal. 2018, 8, 4459–4464.

[45] A. J. M. Miller , J. E. Bercaw , Chem. Commun. 2010, 46, 1709–1711. 10.1039/b925659h 20177624

[46] W. C. Ewing , A. Marchione , D. W. Himmelberger , P. J. Carroll , L. G. Sneddon , J. Am. Chem. Soc. 2011, 133, 17093–17099. 21961893 10.1021/ja207971h

[47] W. R. H. Wright , E. R. Berkeley , L. R. Alden , R. T. Baker , L. G. Sneddon , Chem. Commun. 2011, 47, 3177–3179. 10.1039/c0cc05408a 21283839

[48] C. A. De Albuquerque Pinheiro , C. Roiland , P. Jehan , G. Alcaraz , Angew. Chem. Int. Ed. 2018, 57, 1519–1522; 10.1002/anie.201710293 29206342

[49] C. Marquardt , T. Jurca , K.-C. Schwan , A. Stauber , A. V. Virovets , G. R. Whittell , I. Manners , M. Scheer , Angew. Chem. Int. Ed. 2015, 54, 13782–13786; 10.1002/anie.201507084 PMC4648028 26427911

[50] D. Han , M. Joksch , M. Klahn , A. Spannenberg , H.-J. Drexler , W. Baumann , H. Jiao , R. Knitsch , M. R. Hansen , H. Eckert , T. Beweries , Dalton Trans. 2016, 45, 17697–17704. 27757470 10.1039/c6dt03068h

[51] R. Dallanegra , A. P. M. Robertson , A. B. Chaplin , I. Manners , A. S. Weller , Chem. Commun. 2011, 47, 3763–3765. 10.1039/c0cc05460g 21293798

[52] A. L. Colebatch , A. I. McKay , N. A. Beattie , S. A. Macgregor , A. S. Weller , Eur. J. Inorg. Chem. 2017, 4533–4540.

[53] S. Todisco , L. Luconi , G. Giambastiani , A. Rossin , M. Peruzzini , I. E. Golub , O. A. Filippov , N. V. Belkova , E. S. Shubina , Inorg. Chem. 2017, 56, 4296–4307. 28345899 10.1021/acs.inorgchem.6b02673

[54] A. L. Colebatch , B. W. H. Gilder , G. R. Whittell , N. L. Oldroyd , I. Manners , A. S. Weller , Chem. Eur. J. 2018, 24, 5450–5455. 29504649 10.1002/chem.201800737

[55] N. E. Stubbs , T. Jurca , E. M. Leitao , C. H. Woodall , I. Manners , Chem. Commun. 2013, 49, 9098–9100. 10.1039/c3cc44373f 23982163

[56] Paul has classified amine–borane dehydrogenation catalysts into two categories: type I catalysts, which release one equivalent of H_2_ and form linear polyaminoborane as the main product, and type II, catalysts, which release more than one equivalent of H_2_ and form borazine or polyborazylene. S. Bhunya , P. M. Zimmerman , A. Paul , ACS Catal. 2015, 5, 3478–3493.

[57] A. P. M. Robertson , E. M. Leitao , I. Manners , J. Am. Chem. Soc. 2011, 133, 19322–19325. 22035112 10.1021/ja208752w

[58] A. P. M. Robertson , E. M. Leitao , T. Jurca , M. F. Haddow , H. Helten , G. C. Lloyd-Jones , I. Manners , J. Am. Chem. Soc. 2013, 135, 12670–12683. 23941398 10.1021/ja404247r

[59] G. J. Kubas , Metal Dihydrogen and σ-Bond Complexes, Kluwer Academic, New York, 2001.

[60] M. A. Esteruelas , A. M. López , M. Mora , E. Oñate , ACS Catal. 2015, 5, 187–191.

[61] A. Paul , C. B. Musgrave , Angew. Chem. Int. Ed. 2007, 46, 8153–8156; 10.1002/anie.200702886 17886331

[62] M. Roselló-Merino , J. López-Serrano , S. Conejero , J. Am. Chem. Soc. 2013, 135, 10910–10913. 23822667 10.1021/ja404655v

[63] T. Bligaard , R. M. Bullock , C. T. Campbell , J. G. Chen , B. C. Gates , R. J. Gorte , C. W. Jones , W. D. Jones , J. R. Kitchin , S. L. Scott , ACS Catal. 2016, 6, 2590–2602.

[64] T. Malakar , S. Bhunya , A. Paul , Chem. Eur. J. 2015, 21, 6340–6345. 25787924 10.1002/chem.201405543

[65] A. Ravve , Principles of Polymer Chemistry, Springer, New York, 2012.

[66] C. J. Stevens , R. Dallanegra , A. B. Chaplin , A. S. Weller , S. A. Macgregor , B. Ward , D. McKay , G. Alcaraz , S. Sabo-Etienne , Chem. Eur. J. 2011, 17, 3011–3020. 21322070 10.1002/chem.201002517

[67] D. A. Addy , J. I. Bates , M. J. Kelly , I. M. Riddlestone , S. Aldridge , Organometallics 2013, 32, 1583–1586.

[68] V. Pons , R. T. Baker , N. K. Szymczak , D. J. Heldebrant , J. C. Linehan , M. H. Matus , D. J. Grant , D. A. Dixon , Chem. Commun. 2008, 6597–6599. 10.1039/b809190k 19057791

[69] A. Kumar , H. C. Johnson , T. N. Hooper , A. S. Weller , A. G. Algarra , S. A. Macgregor , Chem. Sci. 2014, 5, 2546.

[70] H. C. Johnson , A. P. M. Robertson , A. B. Chaplin , L. J. Sewell , A. L. Thompson , M. F. Haddow , I. Manners , A. S. Weller , J. Am. Chem. Soc. 2011, 133, 11076–11079. 21699193 10.1021/ja2040738

[71] S. Bhunya , T. Malakar , A. Paul , Chem. Commun. 2014, 50, 5919–5922. 10.1039/c4cc01337a 24763410

[72] J. D. Kim , J. B. P. Soares , G. L. Rempel , Macromol. Rapid Commun. 1998, 19, 197–199.

[73] E. M. Leitao , N. E. Stubbs , A. P. M. Robertson , H. Helten , R. J. Cox , G. C. Lloyd-Jones , I. Manners , J. Am. Chem. Soc. 2012, 134, 16805–16816. 23016922 10.1021/ja307247g

[74] J. R. Turner , D. A. Resendiz-Lara , T. Jurca , A. Schäfer , J. R. Vance , L. Beckett , G. R. Whittell , R. A. Musgrave , H. A. Sparkes , I. Manners , Macromol. Chem. Phys. 2017, 218, 1700120.

[75] T. J. Clark , G. R. Whittell , I. Manners , Inorg. Chem. 2007, 46, 7522–7527. 17663545 10.1021/ic700806b

[76] Y. Kawano , M. Uruichi , M. Shimoi , S. Taki , T. Kawaguchi , T. Kakizawa , H. Ogino , J. Am. Chem. Soc. 2009, 131, 14946–14957. 19772343 10.1021/ja904918u

[77] H. Helten , A. P. M. M. Robertson , A. Staubitz , J. R. Vance , M. F. Haddow , I. Manners , Chem. Eur. J. 2012, 18, 4665–4680. 22392879 10.1002/chem.201103241

[78] N. E. Stubbs , A. Schäfer , A. P. M. Robertson , E. M. Leitao , T. Jurca , H. A. Sparkes , C. H. Woodall , M. F. Haddow , I. Manners , Inorg. Chem. 2015, 54, 10878–10889. 26535961 10.1021/acs.inorgchem.5b01946

[79] D. A. Resendiz-Lara , N. E. Stubbs , M. I. Arz , N. E. Pridmore , H. A. Sparkes , I. Manners , Chem. Commun. 2017, 53, 11701–11704. 10.1039/c7cc07331c 29022601

[80] C. A. Jaska , K. Temple , A. J. Lough , I. Manners , J. Am. Chem. Soc. 2003, 125, 9424–9434. 12889973 10.1021/ja030160l

[81] M. E. Bluhm , M. G. Bradley , R. Butterick , U. Kusari , L. G. Sneddon , J. Am. Chem. Soc. 2006, 128, 7748–7749. 16771483 10.1021/ja062085v

[82] C. Lichtenberg , M. Adelhardt , T. L. Gianetti , K. Meyer , B. De Bruin , H. Grützmacher , ACS Catal. 2015, 5, 6230–6240.

[83] X. Chen , J.-C. Zhao , S. G. Shore , Acc. Chem. Res. 2013, 46, 2666–2675. 24020948 10.1021/ar400099g

[84] J. Li , S. M. Kathmann , H. S. Hu , G. K. Schenter , T. Autrey , M. Gutowski , Inorg. Chem. 2010, 49, 7710–7720. 20701247 10.1021/ic100418a

[85] M. Trose , M. Reiß , F. Reiß , F. Anke , A. Spannenberg , S. Boye , A. Lederer , P. Arndt , T. Beweries , Dalton Trans. 2018, 47, 12858–12862. 30156242 10.1039/c8dt03311k

